# Hypokalemia, Its Contributing Factors and Renal Outcomes in Patients with Chronic Kidney Disease

**DOI:** 10.1371/journal.pone.0067140

**Published:** 2013-07-02

**Authors:** Hsiao-Han Wang, Chi-Chih Hung, Daw-Yang Hwang, Mei-Chuan Kuo, Yi-Wen Chiu, Jer-Ming Chang, Jer-Chia Tsai, Shang-Jyh Hwang, Julian L. Seifter, Hung-Chun Chen

**Affiliations:** 1 Division of Nephrology, Kaohsiung Medical University Hospital, Kaohsiung Medical University, Kaohsiung, Taiwan; 2 Department of Internal Medicine, Kaohsiung Municipal Hsiao-Kang Hospital, Kaohsiung Medical University, Kaohsiung, Taiwan; 3 Faculty of Renal Care, College of Medicine, Kaohsiung Medical University, Kaohsiung, Taiwan; 4 Faculty of Medicine, College of Medicine, Kaohsiung Medical University, Kaohsiung, Taiwan; 5 Renal Division, Department of Medicine, Brigham and Women’s Hospital and Harvard Medical School, Boston, Massachusetts, United States of America; 6 Department of Dermatology, Wan Fang Hospital, Taipei Medical University, Taipei, Taiwan; University of Sao Paulo Medical School, Brazil

## Abstract

**Background:**

In the chronic kidney disease (CKD) population, the impact of serum potassium (sK) on renal outcomes has been controversial. Moreover, the reasons for the potential prognostic value of hypokalemia have not been elucidated.

**Design, Participants & Measurements:**

2500 participants with CKD stage 1–4 in the Integrated CKD care program Kaohsiung for delaying Dialysis (ICKD) prospective observational study were analyzed and followed up for 2.7 years. Generalized additive model was fitted to determine the cutpoints and the U-shape association between sK and end-stage renal disease (ESRD). sK was classified into five groups with the cutpoints of 3.5, 4, 4.5 and 5 mEq/L. Cox proportional hazard regression models predicting the outcomes were used.

**Results:**

The mean age was 62.4 years, mean sK level was 4.2±0.5 mEq/L and average eGFR was 40.6 ml/min per 1.73 m^2^. Female vs male, diuretic use vs. non-use, hypertension, higher eGFR, bicarbonate, CRP and hemoglobin levels significantly correlated with hypokalemia. In patients with lower sK, nephrotic range proteinuria, and hypoalbuminemia were more prevalent but the use of RAS (renin-angiotensin system) inhibitors was less frequent. Hypokalemia was significantly associated with ESRD with hazard ratios (HRs) of 1.82 (95% CI, 1.03–3.22) in sK <3.5mEq/L and 1.67 (95% CI,1.19–2.35) in sK = 3.5–4 mEq/L, respectively, compared with sK = 4.5–5 mEq/L. Hyperkalemia defined as sK >5 mEq/L conferred 1.6-fold (95% CI,1.09–2.34) increased risk of ESRD compared with sK = 4.5–5 mEq/L. Hypokalemia was also associated with rapid decline of renal function defined as eGFR slope below 20% of the distribution range.

**Conclusion:**

In conclusion, both hypokalemia and hyperkalemia are associated with increased risk of ESRD in CKD population. Hypokalemia is related to increased use of diuretics, decreased use of RAS blockade and malnutrition, all of which may impose additive deleterious effects on renal outcomes.

## Introduction

The kidney plays a major role in potassium homeostasis by renal mechanisms that transport and regulate potassium secretion, reabsorption and excretion [1]. Hyperkalemia is a common electrolyte disturbance in patients with chronic kidney disease (CKD) [2]. As eGFR decreases from above 60 to below 20 ml/min/1.73 m^2^, the prevalence of hyperkalemia increases from 2 to 42% [3]. In CKD individuals, a few studies suggested weak links between hyperkalemia and ESRD [4], [5].

Chronic hypokalemia, on the other hand, has been reported to enhance renal cytogenesis and may lead to interstitial scarring and renal insufficiency [6], [7]. Recently, in CKD patients comorbid with and without cardiovascular diseases, the associations between hypokalemia and death as well as ESRD have been proposed [4], [5], [8]. One paper demonstrated hypokalemia was associated with ESRD but this effect was attenuated after adjusting nutritional indices in patients with CKD stage 3 to 5 [4]. Another study suggested hypokalemia was associated with renal progression but the association with hard renal outcome, such as reaching ESRD was unclear [5].

Recent studies have reported common predispositions such as diabetes, high dietary potassium and renin-angiotensin-aldosterone system blockers use for the development of hyperkalemia in pateints with impaired renal functions [9]–[12]. Kaliuretic diuretics such as furosemide and thiazides are common causes of hypokalemia [13], [14]. Diarrhea, vomiting, hyperaldosteronism, magnesium deficiency and potassium redistribution induced by insulin, alkalosis and/or β-adrenergic activation, are all possible prerequisites for hypokalemia [15]. However, studies on hypokalemia and aforementioned associated factors were done mostly in general population. Moreover, the possible complex interplay between hypokalemia, its associating factors and renal outcomes has not been investigated.

In the CKD population, whether sK level is associated with greater risks of renal outcomes has not been clearly defined. Moreover, the verification of the reasons for the potential prognostic value of hypokalemia remains to be elucidated. Thus, we investigated the contributing factors of hypokalemia and whether hyperkalemia or hypokalemia is a risk factor for adverse renal outcomes in patients with CKD stage 1 to 4.

## Methods

### Participants and Measurement

Integrated CKD care program Kaohsiung for delaying Dialysis (ICKD) study was designed as a prospective cohort to investigate the impact of integrated CKD care program on clinical outcomes from a diverse group of CKD stage 1–5 patients. The included population was CKD patients not on renal replacement therapy. The exclusion criterion was acute kidney injury defined as more than 50% decrease in eGFR in three months. The study recruited patients from the nephrology out-patient departments of two hospitals in southern Taiwan. Between November 11, 2002 and May 31, 2009, 3749 patients received integrated CKD care program and were followed up until July 31, 2010. 90 patients were lost for follow-ups in less than 3 months and 1159 stage 5 CKD patients were excluded. Total 2500 subjects with CKD stage 1 to 4 were analyzed. The study protocol was approved by the institutional review board of the Kaohsiung Medical University Hospital (KMUH-IRB-990198). Informed consents have been obtained in written form from patients and all clinical investigation was conducted according to the principles expressed in the Declaration of Helsinki. The patients gave consent for the publication of the clinical details.

At the baseline visit, socio-demographic characteristics, medical history, lifestyle behaviors and current medications were recorded. The medical history was confirmed by doctors’ chart-review. Diabetes mellitus (DM) and hypertension were defined by clinical diagnosis. Cardiovascular disease (CVD) was defined as clinical diagnosis of heart failure, acute or chronic ischemic heart disease, or cerebrovascular disease. Body Mass Index (BMI) was calculated from the baseline enrolled measurement using the formula: weight in kilograms/(height in meters)^2^. Mean blood pressure (MBP) was calculated by the averages of systolic and diastolic BP measured 3 months before and after the enrollment using the formula: 2/3 average systolic BP +1/3 average diastolic BP.

Biochemistry measurements were done on screening visit, baseline visit and then every 3 months as the protocol. The laboratory data from 3 months before baseline visit to 3 months after baseline visit were averaged and analyzed. Six-month averaged sK was used for analysis and was classified into five groups as sK <3.5; K = 3.5–4; K = 4–4.5, K = 4.5–5 and K >5 mEq/L. The cutpoints were defined by generalized additive regression. Serum creatinine, albumin, cholesterol, C-reactive protein (CRP), potassium and phosphate levels were determined by means of the Toshiba TBA C16000 chemistry analyzer. Serum hemoglobin was determined by means of the Sysmex XE-2100 automated hematology analyzer. HbA1C was measured by Primus CLC 385 automated analyzer. Venous blood bicarbonate was measured by AVL Omni 3 blood gas analyzer.

### Quantification of Renal Function and Progression

Kidney function was quantified by using the estimated glomerular filtration rate (eGFR) derived from the simplified Modification of Diet in Renal Disease (MDRD) Study equation. The equation was eGFR ml/min/1.73 m^2^ = 186 × Serum creatinine ^−1.154^ × Age ^−0.203^ × 0.742 (if female) × 1.212 (if black patient). The average eGFR slope (ml/min/1.73 m^2^/yr) for each patient was calculated by linear regression with varying-intercept and varying-slope without co-variates for estimation of the annual change of eGFR.

### Outcomes

Renal outcomes were assessed: end stage renal disease (ESRD) and rapid renal progression. ESRD was defined as the initiation of hemodialysis, peritoneal dialysis or renal transplantation. The initiation of renal replacement therapy was discovered by review of charts or catastrophic cards. Rapid renal progression was defined as an eGFR slope<−6.88 ml/min/1.73 m^2^/yr which was the 20th percentile. Other cut-off values were also applied in sensitivity tests. The timing for ESRD was according to the regulation of Bureau of the National Health Insurance of Taiwan regarding the laboratory data, nutritional status, uremic status and serum creatinine (>6 mg/dL). ESRD was ascertained by reviewing charts and by matching with the database from Taiwan Society of Nephrology.

### Statistical Analysis

Statistic results of baseline characteristics of all subjects were expressed as percentages for categorical variables, mean ± standard deviation (SD) for continuous variables with approximately-normal distribution, and median and interquartile range for continuous variables with skewed distribution.

We conducted both linear regression and generalized additive models (GAM) to investigate the variables associated with sK. GAM were fitted to detect nonlinear effects of continuous covariates (VGAM function of the VGAM package) [16], [17] for sK because GAM were developed for smoothing the effects of covariates in generalized linear models. The selection of variables was based on previous models and also the nutritional and medication variables that we hypothesized.

Cox proportional hazards models were used to evaluate association of variables with time to ESRD. Covariates included in these models were age, gender, DM, CVD, eGFR, Urine protein by dipstick, Angiotensin-converting enzyme inhibitor (ACEI), Angiotensin II receptor blocker (ARB), diuretics, MBP, BMI, HbA1c, log-transformed cholesterol, log-transformed CRP, phosphate, hemoglobin and albumin. Generalized additive models with restricted cubic regression splines were used to explore the functional form of the relationship between sK and ESRD. The knots for sK were at 4.03 and 5.11 mEq/L and we thus divided sK accordingly in the tables. Binary logistic regression analysis was used to assess the relationship between sK and rapid renal progression. Covariates included into these models were the same as those in Cox regression.

Also, the statistical tools for regression diagnostics such as verification of proportional hazards assumption, residual analysis, detection of influential cases, and check for multicollinearity were applied to discover model or data problems. Statistical analysis was performed using the R 2.15.2 software (R Foundation for Statistical Computing, Vienna, Austria) and Statistical Package for Social Sciences version 18.0 for Windows (SPSS Inc., Chicago, IL).

## Results

### Baseline Characteristics of CKD Patients Divided by Serum Potassium Quintiles

**Figure 1:**
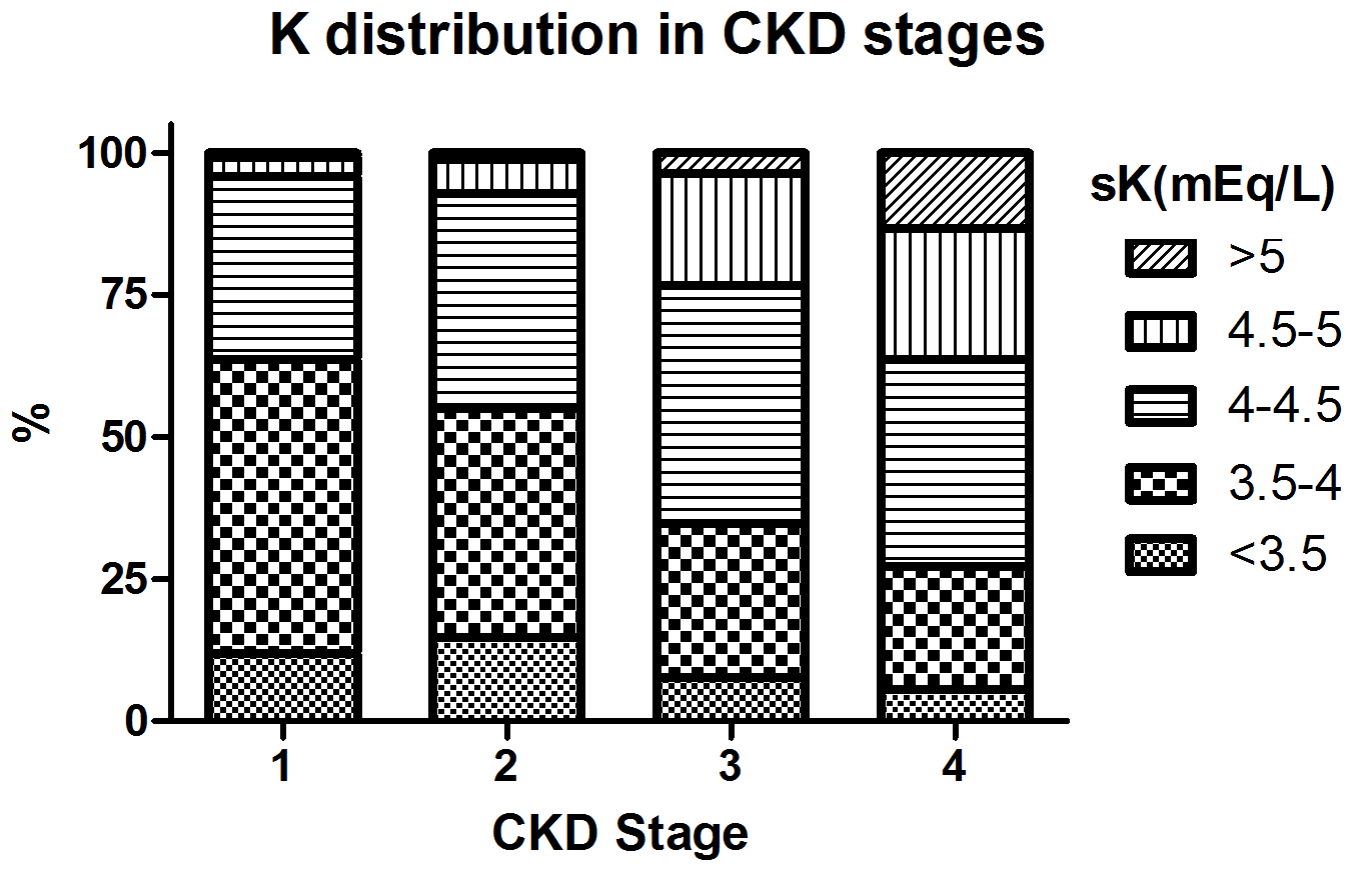
Serum Potassium Distribution by Chronic Kidney Disease (CKD) Stages.

**Figure 2:**
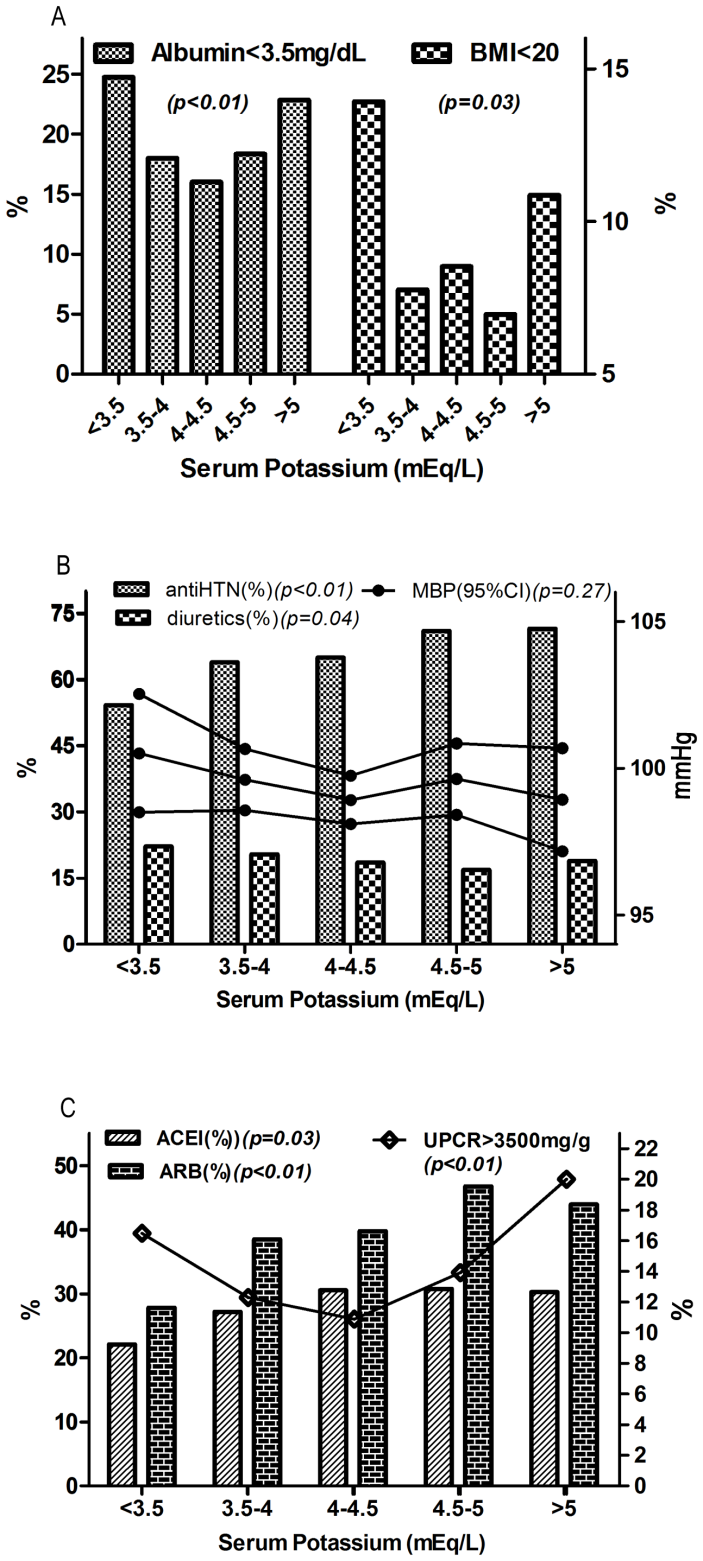
The Relationships between Serum Potassium and Other Predictive Covariates. Figure 2A. Percentages of malnutrition indices including serum albumin lower than 3.5 mg/dL and body mass index (BMI) less than 20 kg/m^2^ across potassium groups. Figure 2B. Proportions of diuretics use (Thiazide and/or Furosemide use), anti-hypertensive (anti-HTN) medication and mean blood pressure (MBP) across potassium groups. Figure 2C. Proportions of ACEI, ARB and proteinuria with nephrotic range across potassium groups. ACEI, angiotensin-converting enzyme inhibitor; ARB, angiotensin II receptor blocker; UPCR, Urine protein-to-creatinine ratio. P-values were generated by chi-square tests.

A total of 2500 participants in CKD stage 1–4 were analyzed during a median 2.7-year of follow-up. Figure 1 displayed the distribution of five sK groups by CKD stage 1–4. There was still 5.6% of participants with sK <3.5 mEq/L in those with CKD stage 4, while there was less than 1% of patients with sK >5 mEq/L in those with CKD stage 1. The majority of the participants were male (64%), had hypertension (61.5%) with a mean age of 62.4±14.5 years, mean sK of 4.2±0.5 mEq/L and mean eGFR of 40.6±23.3 ml/min/1.73 m^2^ (Table 1). Patients with lower sK were more likely to have hypertension, higher eGFR, higher serum hemoglobin, cholesterol and bicarbonate levels, whereas they were less likely to have a history of diabetes, to have higher HbA1c and phosphate levels, in comparison with those with higher sK. Figure 2 further demonstrated the relationship between sK and other important predictive covariates. There were higher proportions of patients presenting with hypoalbuminemia (<3.5 mg/dL) and lower BMI (20 kg/m^2^) in the groups with sK <3.5 and >5mEq/L (Figure 2A).

**Table 1:** Baseline Demographic and Clinical Characteristics.

Variable		All (n = 2500)		sK <3.5 mEq/L (n = 194)		sK = 3.5–4 mEq/L (n = 683)		sK = 4–4.5 mEq/L (n = 974)		sK = 4.5–5 mEq/L (n = 474)		sK >5 mEq/L (n = 175)		*P* for trend	
**Demographics and Medical History**															
Age (years)		62.4±14.5		62.1±14.9		61.3±15.0		62.7±14.1		63.0±14.3		63.8±14.7		0.102	
Gender (female %)		899 (36.0)		73 (37.6)		251 (36.7)		354 (36.3)		163 (34.4)		58 (33.1)		0.234	
Hypertension (%)		1538 (61.5)		131 (67.5)		434 (63.6)		586 (60.2)		281 (59.3)		106 (60.6)		0.038	
Diabetes mellitus (%)		1045 (41.8)		70 (36.1)		260 (38.1)		404 (41.5)		225 (47.5)		86 (49.1)		<0.001	
Cardiovascular disease (%)		564 (22.6)		43 (22.2)		161 (23.6)		206 (21.1)		101 (21.3)		53 (30.3)		0.462	
**Physical Examination**															
BMI (kg/m^2^)		25.1±4.0		25.1±4.2		25.2±3.9		25.0±4.0		25.2±4.1		24.8±4.3		0.578	
MBP (mmHg)		99.4±13.4		100.5±14.2		99.6±13.9		98.9±13.2		99.6±13.5		98.9±11.8		0.279	
**Renal Function Status**															
CKD stage														<0.001	
Stage 1 (%)		118 (4.7)		14 (7.2)		61 (8.9)		38 (3.9)		4 (0.8)		1 (0.6)			
Stage 2 (%)		238 (9.5)		35 (18.0)		96 (14.1)		90 (9.2)		14 (3.0)		90 (9.2)			
Stage 3 (%)		1183 (47.3)		91 (46.9)		319 (46.7)		496 (50.9)		234 (49.4)		496 (50.9)			
Stage 4 (%)		961 (38.4)		54 (27.8)		207 (30.3)		350 (35.9)		222 (46.8)		350 (35.9)			
eGFR (mL/min/1.73 m^2^)		40.6±23.3		47.9±28.2		47.1±28.2		40.5±21.2		33.4±14.5		27.2±12.3		<0.001	
Proteinuria by dipstick (%)															
none	920 (36.8)		77 (39.7)		267 (39.1)		360 (37.0)		163 (34.4)		360 (37.0)		<0.001		
+	652 (26.1)		55 (28.4)		185 (27.1)		262 (26.9)		117 (24.7)		262 (26.9)				
++	481 (19.2)		24 (12.4)		122 (17.9)		202 (20.7)		90 (19.0)		202 (20.7)				
+++∼++++	446 (17.8)		38 (19.6)		108 (15.8)		150 (15.4)		104 (21.9)		150 (15.4)				
**Laboratory Data**															
Albumin (mg/dL)		3.9±0.5		3.8±0.7		3.9±0.6		3.9±0.5		3.9±0.5		3.8±0.5		0.595	
Hemoglobin (mg/dL)		12.2±2.2		12.6±2.3		12.6±2.2		12.3±2.1		11.8±2.2		10.9±1.8		<0.001	
Total cholesterol (mg/dL)		201.4±58.2		212.2±84.2		204.5±62.3		199.2±50.3		197.5±56.0		201.3±52.1		0.022	
CRP (mg/L)		1.0 (0.3−4.7)		1.5 (0.5−4.9)		1.1 (0.4−4.6)		0.9 (0.3−4.0)		1.0 (0.2 − 5.5)		0.9 (0.3 − 4.0)		0.205	
HbA1c (%)		6.7±1.7		6.5±1.8		6.5±1.7		6.6±1.7		7.0±1.8		6.7±1.7		0.007	
Potassium (mEq/L)		4.2±0.5		3.3±0.2		3.8±0.1		4.3±0.1		4.7±0.1		5.3±0.3		<0.001	
Bicarbonate (mEq/L)		23.7±3.7		25.3±3.6		24.4±3.8		23.7±3.4		23.1±3.7		21.3±3.7		<0.001	
Phosphate (mg/dL)		3.9±0.8		3.7±0.9		3.8±0.8		3.8±0.8		4.1±0.8		4.4±0.9		<0.001	
**Surrogate endpoints**															
eGFR slope (mL/min/1.73 m^2^/yr)		−1.8 (−5.6−0.7)		−1.0 (−7.9−1.7)		−1.5 (−5.3−1.4)		−1.8 (−5.2−0.6)		−1.9 (−5.1 − 0.3)		−3.5 (−7.8 − −1.0)		<0.001	
Rapid renal progression*		492 (20.0)		50 (26.2)		129 (19.3)		173 (18.1)		92 (19.5)		48 (27.9)		0.765	
Proteinuria increment#		255.0±489.7		357.8±830.8		278.3±537.3		216.0±376.8		225.0±349.5		347.8±622.1		0.489	
**Primary endpoints**															
ESRD (%)		299 (12.0)		16 (8.2)		69 (10.1)		93 (9.5)		69 (14.6)		52 (29.7)		<0.001	
Rate per 1000 person-Years		43		37		39		33		47		109			

Data expressed as mean ± standard deviation, median (interquartile range) or percentage.

BMI, body mass index; MBP, mean blood pressure; CKD, chronic kidney disease; eGFR, estimated glomerular filtration rate; CRP, C-reactive protein; HbA1c, glycated hemoglobin; UPCR, Urine protein-to-creatinine ratio; ESRD, End Stage Renal Disease.

Comparisons are made by ANOVA or the chi-square test.

* eGFR slope less than −6.88 mL/min/1.73 m^2^/yr (%).

# Urine protein-to-creatinine (mg/g).

In total participants, ESRD rate was 43 per 1000 person-years (Table 1). There was a trend of greater crude rates of ESRD and steeper decline in eGFR in groups with higher sK. There were no significant univariate linear relationships between sK groups and rapid renal progression and increment of proteinuria respectively. However, greater increase of proteinuria and higher proportion of participants with rapid renal progression were observed in the groups with sK <3.5 mEq/L and sK >5 mEq/L with p for Chi-Square as p = 0.009 and p<0.001 respectively.

The percentages of patients receiving ACEI, ARB, oral hypoglycemic agents and insulin were higher in those with more elevated sK (Table 2). The proportions of other medication use such as furosemide, thiazide, phosphate binder and β-blocker did not show significant trends among groups. Despite similar MBP across five groups of sK, the use of anti-hypertensive medication was less frequent while the use of diuretics was more prevalent as sK decreased (Figure 2B). The prevalence of nephrotic range proteinuria was the highest in the groups with sK <3.5 and >5 mEq/L but the use of RAS inhibitors was the least in sK <3.5 mEq/L (Figure 2C).

**Table 2:** Use of Medications.

	All (n = 2500)	sK <3.5 mEq/L (n = 194)	sK = 3.5−4 mEq/L (n = 683)	sK = 4−4.5 mEq/L (n = 974)	sK = 4.5−5 mEq/L (n = 474)	sK >5 mEq/L(n = 175)	*P* for trend
ACEI (%)	726 (29.0%)	43 (22.2)	186 (27.2)	298 (30.6)	146 (30.8)	53 (30.3)	0.026
ARB (%)	1004 (40.2%)	54 (27.8)	263 (38.5)	388 (39.8)	222 (46.8)	77 (44.0)	<0.001
Furosemide (%)	313 (12.5%)	29 (14.9)	90 (13.2)	120 (12.3)	52 (11.0)	22 (12.6)	0.067
Thiazide (%)	237 (9.5%)	24 (12.4)	72 (10.5)	87 (8.9)	36 (7.6)	18 (10.3)	0.051
Oral hypoglycemic agents (%)	725 (29.0%)	42 (21.6)	165 (24.2)	271 (27.8)	180 (38.0)	67 (38.3)	<0.001
Insulin (%)	153 (6.1%)	7 (3.6)	17 (2.5)	61 (6.3)	50 (10.5)	18 (10.3)	<0.001
Phosphate binders (%)	310 (12.4%)	16 (8.2)	92 (13.5)	112 (11.5)	63 (13.3)	27 (15.4)	0.161
β-blocker (%)	571 (22.8%)	43 (22.2)	169 (24.7)	210 (21.6)	118 (24.9)	31 (17.7)	0.386

Data expressed as mean ± standard deviation, median (interquartile range) or percentage.

ACEI, angiotensin-converting enzyme inhibitor; ARB, angiotensin II receptor blocker.

All the univariate significant and non-significant but relevant covariates were tested in multivariate linear regression for sK (Table 3). Non-linear effects of continuous variables were tested by generalized additive models (Table S1 and Figure S1). sK was negatively correlated with eGFR, MBP, bicarbonate, CRP and hemoglobin and positively correlated with HbA1c and albumin. The use of ACEI, ARB, and insulin vs those non-users were associated with higher sK. Female vs. male and diuretics users vs. non-users were associated with lower sK (Table 3 and Table S1).

**Table 3:** Linear Regression of Serum Potassium.

Multivariate linear regression			
Variables	Beta coefficient	95% CI Beta coefficient	*p*
constant	4.83		
Gender (female)	−0.13	−0.18 to −0.09	<0.001
GFR per 10 mL/min/1.73 m^2^	−0.03	−0.04 to −0.02	<0.001
ACEI user vs non-user	0.05	0.005 to 0.09	0.026
ARB user vs non-user	0.06	0.02 to 0.10	0.007
Diuretics use vs non-user	−0.13	−0.18 to −0.08	<0.001
Bicarbonate (mEq/L)	−0.03	−0.03 to −0.02	<0.001
Phosphorus (mg/dL)	0.08	0.05 to 0.10	<0.001
Log-transformed CRP	−0.05	−0.07 to −0.03	<0.001
HbA1c (%)			
In total population	0.02	0.01 to 0.04	0.001
In HbA1c <10% group*	0.044	0.025 to 0.064	<0.001
In HbA1c ≧10% group*	−0.081	−0.141 to −0.021	0.008
Hemoglobin (g/dL)	−0.03	−0.04 to −0.01	<0.001
Albumin (g/dL)			
In total population	0.09	0.05 to 0.14	<0.001
In albumin <4 g/dL group*	0.161	0.096 to 0.225	<0.001
In albumin ≧4 g/dL group*	−0.156	−0.288 to −0.024	0.021
MBP (mmHg)	−0.002	−0.004 to −0.001	0.002
Insulin user vs non-user	0.15	0.07 to 0.24	<0.001

ACEI, angiotensin-converting enzyme inhibitor; ARB, angiotensin II receptor blocker.

MBP, mean blood pressure; eGFR, estimated glomerular filtration rate; CRP, C-reactive protein; HbA1c, glycated hemoglobin; UPCR, Urine protein-to-creatinine ratio.

All relevant variables were tested. Variables with significance were presented. * segmental linear regression with the same variables.

### Potassium and Renal Replacement Therapy

**Figure 3:**
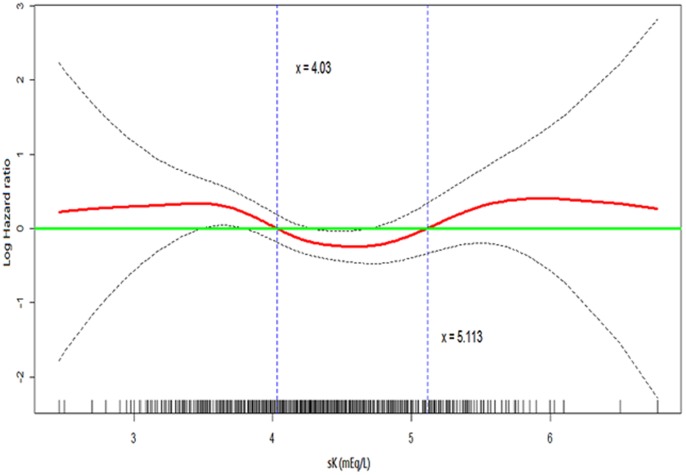
Restricted Cubic Spline Regression Plot of the U-shape Association between sK and the Risk for End Stage Renal Disease. Covariates included in the model were the same as the Cox regression in Table 4. Serum potassium (sK), body mass index, mean blood pressure and C-reactive protein were treated as restricted cubic spline functions. The solid line represents the log transformed multivariable-adjusted hazard ratio of ESRD. The dashed lines indicate the 95% confidence intervals. sK below 4.03 and above 5.11 mEq/L were associated with higher hazard (log hazard ratio >0). Tick marks on the x-axis indicate individual observations at corresponding levels of sK.

There were 299 patients (12%) reaching ESRD during a median 2.7-year of follow-up. To explore the non-linear association of sK and ESRD, we fitted a generalized additive model with a restricted cubic spline regression (Figure 3). The result revealed a U-shaped association, with increased risk of ESRD when sK <4.03 mEq/L and sK >5.11 mEq/L. Consistent with these associations, when compared to sK = 4.5–5mEq/L, both sK <3.5 mEq/L and sK = 3.5–4 mEq/L were significantly associated with 82% (HR 1.82; CI 95% 1.03–3.22; p = 0.041) and 67% (HR 1.67; CI 95% 1.19–2.35; p = 0.003) excess risk of ESRD, respectively in fully-adjusted Cox regression (Table 4). sK >5mEq/L was also associated with increased risk of becoming ESRD with HR of 1.60 (CI 95% 1.09–2.34).

**Table 4:** Association of Categorical Potassium with ESRD and Rapid Renal Progression in Full-Adjusted Model.

	ESRD		Rapid renal progression*	
Risk factors	HR (95% CI)	*p*	OR (95% CI)	*p*
constant	2.71		3.04	
Diabetes mellitus	1.57 (1.19 to 2.08)	0.001	1.30 (1.00 to 1.69)	0.047
eGFR ml/min/1.73 m^2^	0.92 (0.90 to 0.93)	<0.001	1.02 (1.01 to 1.02)	<0.001
Proteinuria by dipstick				
−	1(reference)		1(reference)	
+	2.06 (1.26 to 3.35)	0.004	1.44 (1.02 to 2.02)	0.036
++	4.11 (2.61 to 6.47)	<0.001	2.86 (2.04 to 3.99)	<0.001
+++∼++++	7.50 (4.73 to 11.88)	<0.001	4.83 (3.34 to 6.95)	<0.001
ARB user vs non-user	0.88 (0.69 to 1.12)	0.312	0.69 (0.55 to 0.89)	0.004
Diuretics user vs non-user	1.44 (1.07 to 1.93)	0.015	1.14 (0.85 to 1.53)	0.386
Potassium				
sK <3.5 mEq/L	1.82 (1.03 to 3.22)	0.041	1.69 (1.06 to 2.70)	0.027
sK = 3.5−4 mEq/L	1.67 (1.19 to 2.35)	0.003	1.18 (0.83 to 1.66)	0.364
sK = 4−4.5 mEq/L	1.23 (0.89 to 1.70)	0.205	1.12 (0.82 to 1.55)	0.470
sK = 4.5−5 mEq/L	1 (reference)		1 (reference)	
sK >5 mEq/L	1.60 (1.09 to 2.34)	0.016	1.39 (0.89 to 2.20)	0.149
Body mass index (kg/m^2^)	0.96 (0.94 to 0.99)	0.016	0.97 (0.95 to 1.00)	0.072
MBP (mmHg)	1.01 (1.00 to 1.02)	0.019	1.01 (1.01 to 1.02)	0.001
Phosphorus (mg/dL)	1.36 (1.20 to 1.54)	<0.001	1.21 (1.05 to 1.39)	0.008
HbA1c (%)	1.09 (1.02 to 1.17)	0.013	1.10 (1.03 to 1.18)	0.004
Hemoglobin (g/dL)	0.85 (0.79 to 0.92)	<0.001	0.82 (0.77 to 0.88)	<0.001
Albumin (g/dL)	0.45 (0.36 to 0.57)	<0.001	0.61 (0.49 to 0.77)	<0.001

* eGFR slope less than −6.88 ml/min/1.73 m^2^/yr.

Values expressed as hazard ratio (95% confidence interval) [HR (95% CI)] and odds ratio (95% confidence interval) [OR (95% CI)].

Full-adjusted model included age, gender, DM, CVD, eGFR, Urine protein by dipstick, ACEI, ARB, diuretics, MBP, BMI, HbA1c, log-transformed cholesterol, log-transformed CRP, phosphate, hemoglobin and albumin. Variables with significance were presented.

Other risk factors for ESRD revealed in the multivariate Cox regression analysis included diabetes, decreased eGFR, diuretic use, higher MBP, proteinuria (by dipstick or by urine protein-to-creatinine ratio (UPCR)), hyperphosphatemia, higher HbA1c, lower BMI, anemia and hypoalbuminemia (Table 4). We also tested the proportional hazard assumption and linearity assumption. The results were shown in Supplementary Table 2. Three variables including gender, albumin and bicarbonate were against proportional hazard assumption and were treated by stratification. Four variables including serum potassium, BMI, mean blood pressure and C-reactive protein had non-linear relationships with ESRD (Table S2 and Figure S2). An alternative model with UPCR showed similar results (data not shown).

### Subgroup Analysis for Hypokalemia and Renal Replacement Therapy

The association between the sK <3.5mEq/L and ESRD was significant in patients with ARB use (HR 3.14; 1.36–7.24; p<0.001), but not in those without (HR 1.32; 0.58–2.97; p = 0.508; p for interaction, 0.041). The other pre-specified subgroups did not show significant interaction.

### Potassium and Rapid Renal Progression

The median eGFR slope was −1.8 ml/min/1.73 m^2^/yr. There were 492 participants (20%) experiencing eGFR slope less than −6.88 ml/min/1.73 m^2^/yr. Serum K lower than 3.5 mEq/L was significantly associated with faster yearly decline in eGFR with odds ratio (OR) of 1.69 (CI 95% 1.06–2.70; p = 0.027) compared with sK = 4.5–5 mEq/L after full adjustment (Table 4). On the other hand, sK >5mEq/L didn’t show significant association with rapid progression of renal function. Additional risk factors for rapid renal progression included diabetes, higher MBP, less ARB use, proteinuria, hyperphosphatemia, higher HbA1c, anemia and hypoalbuminemia.

## Discussion

In our cohort, we examined the associations between sK, its associating factors and renal outcomes in 2500 patients with CKD stage 1–4. We demonstrated that diuretic use vs. non-use, hypertension, higher bicarbonate, hemoglobin and CRP levels significantly correlated with hypokalemia. The use of ACEI, ARB, and insulin vs. those non-users, higher HbA1c and albumin levels were associated with higher sK. The association of sK with rapid renal progression and ESRD was U shape. Hypokalemia defined as <4 mEq/L and hyperkalemia as >5 mEq/L in our cohort were significantly associated with greater risk of ESRD.

Our results found hypokalemia to be a significant indicator of renal progression in CKD evidenced by 82% increased risk of reaching ESRD, rapid annual decline in eGFR and increase of proteinuria in CKD population. Our results are in accordance with a study of 1227 males with CKD by Kovesdy et al. They found that a sK <3.6 mEq/L was associated with more severe progression (–0.23 ml/min/1.73 m^2^/yr, p = 0.002) compared to sK = 3.6–5.5 [5]. On the contrary, Saran et al. reported that the association between hypokalemia (<4 mEq/L) and ESRD disappeared after adjustment for serum albumin. In our studies, we proposed that insufficient anti-hypertensive medication, increased diuretic use, insufficient RAS inhibition and malnutrition associated with hypokalemia could be related with worse outcomes.

We propose the following as plausible mechanisms: First, less use of anti-hypertensive medication despite similar BP and greater diuretic use were associated with hypokalemia (Fig. 2B, and Table 3). Diuretics are often used in patients with heart and/or renal disease with volume overload. However, chronic diuretic use has been reported to be associated with higher long-term mortality and hospitalization for HF [18]. Our study (Table 4) as well as clinical studies and animal models have suggested a correlation between diuretic use and progression of renal disease [19]. Thiazides have been proposed to associate with renal injury via the induction of hypokalemia, metabolic abnormalities, and volume depletion and to lead to additional focal glomerular damage not observed in states of equivalent hypokalemia in the absence of diuretics [20].

Second, inadequate RAS inhibition related to either insufficient RAS blockade or resistance to RAS inhibitors may be related to hypokalemia. Insufficient RAS blockage was suggested by a greater extent of proteinuria and less use of ACEI/ARB in the hypokalemic population (Fig. 2C). There is well-established evidence that RAS inhibitors (ACEI or ARB) have beneficial effects on renal outcomes in both diabetic kidney disease and non-diabetic patients with proteinuria [21], [22]. However, in the subgroup of ARB use in our cohort, hypokalemia was still associated with increased risk of ESRD. Previous studies had shown that ACE insertion/deletion (I/D) polymorphism contributed to the variability in ACEI treatment response and sodium-potassium balance [23]–[25]. The Antihypertensive and Lipid-Lowering Treatment to Prevent Heart Attack Trial (ALLHAT) reported the associations of mortality with new-onset hypokalemia (<3.5 mEq/L) and hyperkalemia (>5.4 mEq/L) determined at year-1 of treatment with chlorthalidone, lisinopril or amlodipine [26]. The risk of mortality among participants who developed hypokalemia by year 1 in the Lisinopril group was the highest (HR 3.825; CI: 2.26–6.44; P<0.001) than in either of the other 2 groups. From our result and previous reports, hypokalemia might be related to poor response to RAS blockade.

Third, hypoalbuminemia and lower BMI were associated with hypokalemia (Fig. 2A, Table 3). Hypoalbuminemia, as a marker of both nutritional and inflammatory status was associated with an increased rate of mortality in the population with renal disease [27]–[29]. Hypokalemia might also be a surrogate marker of malnutrition and correlated with hypoalbuminemia in both peritoneal and hemodialysis patients [30]–[32]. Both hypoalbuminemia and hypokalemia remained significant predictors of worse renal outcomes when included simultaneously in the Cox regression model (Table 4). These findings may point out that hypokalemia is an independent risk factor for CKD progression and indicates additional prognostic importance of hypoalbuminemia.

Significant correlations between hypokalemia and alkalosis and hypertension respectively were observed (Table 3). Hypokalemia related augmented ammoniagenesis and subsequent renal injury has been documented [33]. Hypokalemia was also reported to stimulate renin and angiotensin II despite direct suppression of aldosterone synthesis leading to salt-sensitive hypertension, intrarenal vasoconstriction and ischemia [34]–[37]. Other mechanisms mediating renal injury in hypokalemia such as increased inflammatory mediators, oxidative stress [38] and impaired angiogenesis [39] have been reported. Human and animal pathologic studies also demonstrated that prolonged hypokalemia was accompanied by development of multiple renal medullary cysts, tubular degeneration, marked interstitial fibrosis and intense macrophage infiltration eventually leading to the impairment of renal function [6], [40].

Hyperkalemia, beginning with serum values greater than >5 mEq//L in our cohort was associated with higher risk of ESRD. Recent post-hoc analysis of the RENAAL trial of Type 2 diabetes mellitus, by De Zeeuw et al. reported that increased sK ≥5 mEq/L after treatment with Losartan was associated with increased risk of ESRD. One potential mechanism could be the vicious cycle of increasing aldosterone levels in response to sustained hyperkalemia [41]. Aldosterone has been proposed to cause renal progression through both hemodynamic effects and direct cellular actions as it promotes endothelial dysfunction, facilitates thrombosis, reduces vascular compliance and causes myocardial and vascular fibrosis [42], [43].

Our study has limitations to be considered. As an observational cohort, our ability to elucidate the definite causal links was limited. The variation of sK levels affected by nutritional status or medication use during the follow-up period could confound the results. However, the average sK level is a more precise description of baseline status than the single measurement mostly used in published studies. Even with markers of nutritional status such as serum albumin and BMI, lack of records of dietary intake might still confound the findings. Only a small group of patients in the sK <3.5 group had rapid progression (26.2%) and incident ESRD (8.2%). These patients also presented heavier proteinuria at baseline. We attempted to account for the differences in baseline proteinura in our adjusted models. However, residual confounding by factors related to the underlying kidney diseases in these patients might still remain. This also limits our ability to make generalizations about the relationships between hypokalemia and renal outcomes. Finally, the follow-up period of 2.7 years could be relatively short for the assessment of progression to ESRD, especially for our study participants with higher eGFR. However, other indicators of renal progression such as the eGFR slope and proteinuria were examined and all pointed to the associations between worse renal outcomes and hypo- and hyperkalemia.

In conclusion, both hypokalemia and hyperkalemia are associated with elevated risk of developing ESRD in a CKD population. Hypokalemia is related to increased use of diuretics, decreased use of RAS blockade and malnutrition, all of which may impose additive deleterious effects on renal outcomes. Clinical implication may be drawn from our study that sK should be routinely evaluated and special attention given for sK between 3.5–4 mEq/L, generally considered as low “normal”. However, further clinical trials are required to determine if interventions aimed at achieving optimal sK level will slow the progression towards ESRD.

## Supporting Information

Figure S1 **Restricted Cubic Spline Regression Model of the Non-linear Effect of BMI, HbA1c and Albumin on sK.** A. Body Mass Index (BMI) below 16.4 and above 36.1 kg/m^2^ were associated with higher sK. Tick marks on the x-axis indicate individual observations at corresponding levels of BMI. The solid line represents the log transformed predictive value of serum potassium (sK). Same annotations were used for Figure S1B and S1C. B. HbA1c below 6.69 and above 12.79% were associated with higher sK. C. Albumin below 3.68 and above 4.56 mg/dL were associated with higher sK.
(TIF)

Figure S2 **Restricted Cubic Spline Regression Model of the Hazard Ratio of sK, BMI, MBP and CRP for End Stage Renal Disease.** A. Serum potassium (sK) below 4.03 and above 5.11 mEq/L were associated with higher hazard. Tick marks on the x-axis indicate individual observations at corresponding levels of sK. The solid line represents the log transformed multivariable-adjusted hazard ratio of ESRD. Same annotations were used for Figure S2B, S2C and S2D. B. Body Mass Index (BMI) below 23.36 and between 29.68 and 39.49 kg/m^2^ were associated with higher hazard for ESRD. C. Mean blood pressure (MBP) below 72.27 and between 101.16 and 127.66 mmHg were associated with higher hazard for ESRD. D. C-reactive protein (CRP) below 39.36 mg/L was associated with higher hazard for ESRD.
(TIF)

Table S1 (DOC)

Table S2 (DOC)
