# Supplementary figures and images for: Hypokalemia, Its Contributing Factors and Renal Outcomes in Patients with Chronic Kidney Disease
Source: PLoS One. 2013 Jul 2;8(7):e67140. doi: 10.1371/journal.pone.0067140 (PMC3699540; doi:10.1371/journal.pone.0067140)

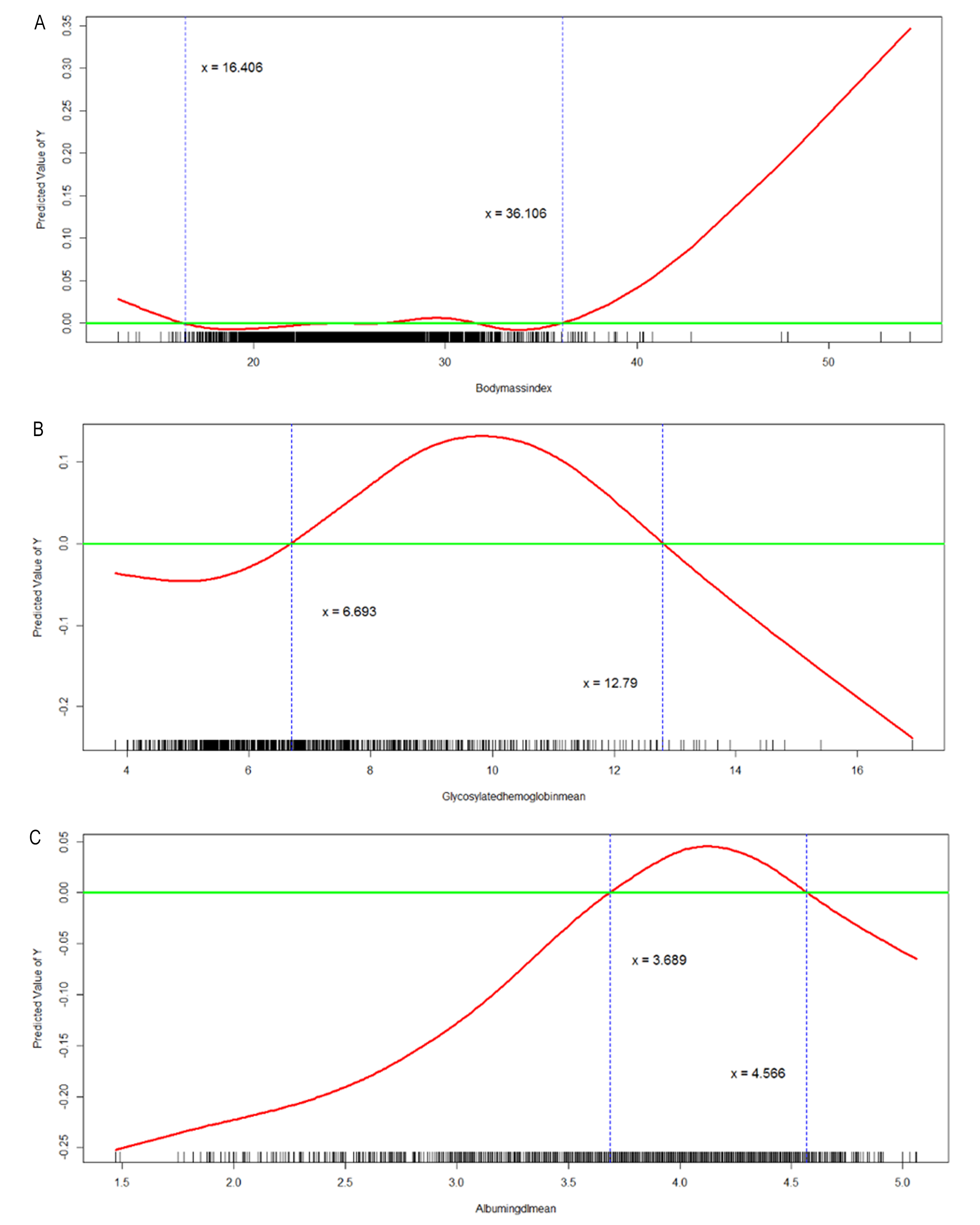

Supplement: Figure S1 — Restricted Cubic Spline Regression Model of the Non-linear Effect of BMI, HbA1c and Albumin on sK. A. Body Mass Index (BMI) below 16.4 and above 36.1 kg/m2 were associated with higher sK. Tick marks on the x-axis indicate individual observations at corresponding levels of BMI. The solid line represents the log transformed predictive value of serum potassium (sK). Same annotations were used for Figure S1B and S1C. B. HbA1c below 6.69 and above 12.79% were associated with higher sK. C. Albumin below 3.68 and above 4.56 mg/dL were associated with higher sK. (TIF) [file pone.0067140.s001.tif]

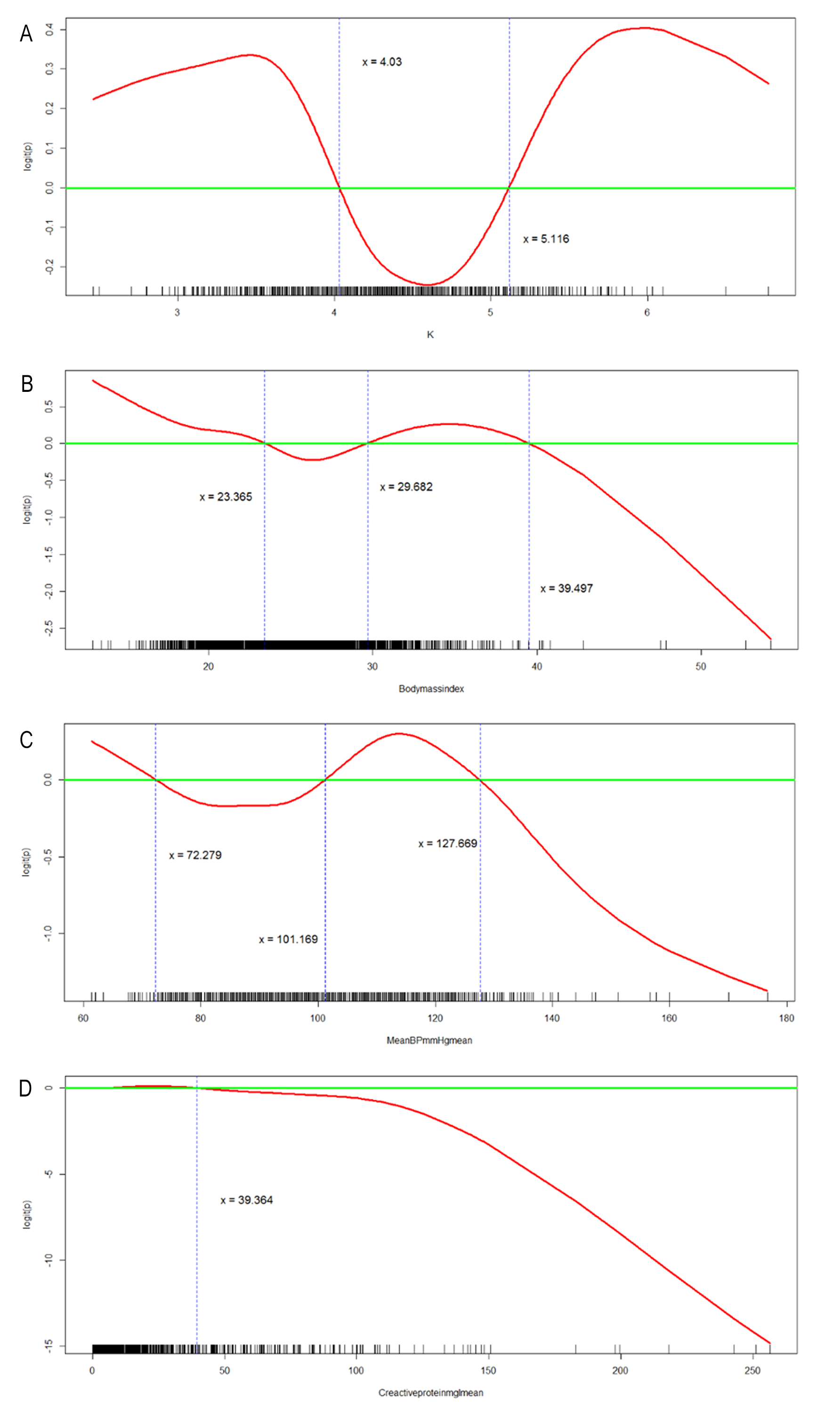

Supplement: Figure S2 — Restricted Cubic Spline Regression Model of the Hazard Ratio of sK, BMI, MBP and CRP for End Stage Renal Disease. A. Serum potassium (sK) below 4.03 and above 5.11 mEq/L were associated with higher hazard. Tick marks on the x-axis indicate individual observations at corresponding levels of sK. The solid line represents the log transformed multivariable-adjusted hazard ratio of ESRD. Same annotations were used for Figure S2B, S2C and S2D. B. Body Mass Index (BMI) below 23.36 and between 29.68 and 39.49 kg/m2 were associated with higher hazard for ESRD. C. Mean blood pressure (MBP) below 72.27 and between 101.16 and 127.66 mmHg were associated with higher hazard for ESRD. D. C-reactive protein (CRP) below 39.36 mg/L was associated with higher hazard for ESRD. (TIF) [file pone.0067140.s002.tif]
