# Supplementary material for: Hypokalemia, Its Contributing Factors and Renal Outcomes in Patients with Chronic Kidney Disease
Source: PLoS One. 2013 Jul 2;8(7):e67140. doi: 10.1371/journal.pone.0067140 (PMC3699540; doi:10.1371/journal.pone.0067140)
Supplement: Table S1 — (DOC) [file pone.0067140.s003.doc]

**Table S1. Generalized Additive Regression for Serum Potassium**

| Variables | Estimate coefficient | 95% CI | *p* |
| --- | --- | --- | --- |
| Constant | 4.84 |  |  |
| Age (years) | -0.001 | -0.002 to 0.001 | 0.665 |
| Gender (female) | -0.13 | -0.17 to -0.09 | <0.001 |
| GFR per 10 mL/min/1.73 m^2^ | -0.03 | -0.04 to -0.03 | <0.001 |
| Diabetes mellitus | -0.02 | -0.07 to 0.03 | 0.526 |
| Cardiovascular disease | -0.01 | -0.06 to 0.03 | 0.581 |
| ACEI user vs non-user | 0.05 | 0.01 to 0.09 | 0.025 |
| ARB user vs non-user | 0.07 | 0.02 to 0.12 | <0.001 |
| Diuretics user vs non-user | -0.12 | -0.18 to -0.08 | <0.001 |
| Body mass index(kg /m^2^) | See supplementary Figure 1a | | |
| Bicarbonate (mEq/L) | -0.03 | -0.04 to -0.02 | <0.001 |
| Phosphorus (mg/dL) | 0.06 | 0.03 to 0.09 | 0.001 |
| Log-transformed CRP | -0.06 | -0.08 to -0.03 | <0.001 |
| HbA1c (%) | See supplementary Figure 1b | | |
| Hemoglobin (g/dL) | -0.02 | -0.04 to -0.01 | <0.001 |
| Albumin (g/dL) | See supplementary Figure 1c | | |
| MBP (mmHg) | -0.001 | -0.003 to -0.001 | 0.043 |
| Insulin user vs non-user | 0.15 | 0.07 to 0.24 | <0.001 |
| Oral hypoglycemic agents user vs non-user | 0.02 | -0.03 to 0.07 | 0.406 |
| Protein by dipstick (+) | -0.08 | -0.13 to -0.04 | 0.001 |
| Protein by dipstick (++) | 0.03 | -0.02 to 0.09 | 0.132 |
| Protein by dipstick (+++) | 0.03 | -0.02 to 0.09 | 0.142 |

Adjusted R square = 0.1787
