# Supplementary material for: Hypokalemia, Its Contributing Factors and Renal Outcomes in Patients with Chronic Kidney Disease
Source: PLoS One. 2013 Jul 2;8(7):e67140. doi: 10.1371/journal.pone.0067140 (PMC3699540; doi:10.1371/journal.pone.0067140)
Supplement: Table S2 — (DOC) [file pone.0067140.s004.doc]

**Table S2. Cox Regression Analysis with Restricted Cubic Spline for End Stage Renal Disease**

|  | ESRD |  |
| --- | --- | --- |
| Risk factors | HR (95% CI) | p |
| Diabetes mellitus | 1.58 (1.25 to 2.16) | <0.001 |
| Cardiovascular disease | 0.96 (0.77 to 1.26) | 0.373 |
| eGFR ml/min/1.73 m^2^ | 0.93 (0.92 to 0.94) | <0.001 |
| Proteinuria by dipstick (0-3) | 1.99 (1.75 to 2.27) | <0.001 |
| ACEI user vs non-user | 1.06 (0.87 to 1.24) | 0.531 |
| ARB user vs non-user | 0.93 (0.79 to 1.11) | 0.293 |
| Diuretics user vs non-user | 1.58 (1.20 to 1.86) | 0.001 |
| sK | See supplementary Figure 2a | |
| Log-transformed cholesterol | 1.42 (0.67 to 3.51) | 0.362 |
| Body mass index(m^2^/kg) | See supplementary Figure 2b | |
| MBP (mmHg) | See supplementary Figure 2c  0.021 | |
| Phosphorus (mg/dL) | 1.32 (1.18 to 1.50) | <0.001 |
| HbA1c (%) | 1.07 (1.00 to 1.16) | 0.054 |
| Log-transformed CRP | See supplementary Figure 2d  0.864 | |
| Hemoglobin (g/dL) | 0.85 (0.79 to 0.91) | <0.001 |

Stratified by albumin, gender and bicarbonate.

Adjusted R square = 0.2762
